# Comparing cellular bone matrices for posterolateral spinal fusion in a rat model

**DOI:** 10.1002/jsp2.1084

**Published:** 2020-03-15

**Authors:** Cliff Lin, Nianli Zhang, Erik I. Waldorff, Paolo Punsalan, David Wang, Eric Semler, James T. Ryaby, Jung Yoo, Brian Johnstone

**Affiliations:** ^1^ Department of Orthopaedics and Rehabilitation Oregon Health & Science University Portland Oregon USA; ^2^ Orthofix Medical Inc Lewisville Texas USA; ^3^ MTF Biologics Edison New Jersey USA

**Keywords:** allograft, athymic rat, biologic therapies, bone graft substitutes, cell‐based therapy, cellular bone matrices, pre‐clinical models, spinal fusion

## Abstract

**Introduction:**

Cellular bone matrices (CBM) are allograft products that provide three components essential to new bone formation: an osteoconductive scaffold, extracellular growth factors for cell proliferation and differentiation, and viable cells with osteogenic potential. This is an emerging technology being applied to augment spinal fusion procedures as an alternative to autografts.

**Methods:**

We aim to compare the ability of six commercially‐available human CBMs (Trinity ELITE®, ViviGen®, Cellentra®, Osteocel® Pro, Bio4® and Map3®) to form a stable spinal fusion using an athymic rat model of posterolateral fusion. Iliac crest bone from syngeneic rats was used as a control to approximate the human gold standard. The allografts were implanted at L4‐5 according to vendor specifications in male athymic rats, with 15 rats in each group. MicroCT scans were performed at 48 hours and 6 weeks post‐implantation. The rats were euthanized 6 weeks after surgery and the lumbar spines were harvested for X‐ray, manual palpation and histology analysis by blinded reviewers.

**Results:**

By manual palpation, five of 15 rats of the syngeneic bone group were fused at 6 weeks. While Trinity ELITE had eight of 15 and Cellentra 11 of 15 rats with stable fusion, only 2 of 15 of ViviGen‐implanted spines were fused and zero of 15 of the Osteocel Pro, Bio4 and Map3 produced stable fusion. MicroCT analysis indicated that total bone volume increased from day 0 to week 6 for all groups except syngeneic bone group. Trinity ELITE (65%) and Cellentra (73%) had significantly greater bone volume increases over all other implants, which was consistent with the histological analysis.

**Conclusion:**

Trinity ELITE and Cellentra were significantly better than other implants at forming new bone and achieving spinal fusion in this rat model at week 6. These results suggest that there may be large differences in the ability of different CBMs to elicit a successful fusion in the posterolateral spine.

## Graphical abstract

We aim to compare the ability of six commercially‐available human CBMs (Trinity ELITE, ViviGen, Cellentra, Osteocel Pro, Bio4 and Map3) to form a stable spinal fusion using an athymic rat model of posterolateral fusion. Our results showed that Trinity ELITE and Cellentra were significantly better than other implants at forming new bone and achieving spinal fusion in this rat model at week 6.

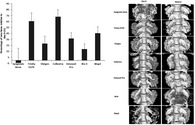

**Abbreviations**
ACDF: anterior cervical discectomy and fusion
ALIF: anterior lumbar interbody fusion
BMD: bone mineral density
CBM: cellular bone matrices
DBM: demineralized bone matrices
MAPC: multipotent adult progenitor‐class cells
MSC: mesenchymal stem cells
NDI: neck disability index
ROI: region of interest
TBV: total bone volume
VAS: visual analog scale
XLIF: lateral lumbar interbody fusion

## 1 INTRODUCTION

Spinal fusion is a widely accepted procedure used to treat a variety of spinal pathologies including spondylolisthesis, deformity, trauma and oncologic disorders. Establishing a solid bony fusion is key to the success of these procedures. Autologous iliac crest bone harvest has traditionally been the gold standard bone graft due to its osteogenic, osteoinductive and osteoconductive properties. However, due to complications associated with limited availability and donor site morbidity,^1^, ^2^, ^3^ there has more recently been an increased use of allogeneic or synthetic bone graft substitutes, some of which are augmented with osteoinductive growth factors, such as bone morphogenetic proteins.^4^, ^5^

When harvested and implanted as part of the same surgical procedure, autologous bone provides the three components essential to new bone formation: an osteoconductive scaffold, extracellular growth factors for cell proliferation and differentiation, and viable cells with osteogenic potential.^6^ Recent technological developments in the processing and preservation of allogeneic bone grafts have made it possible to offer alternative solutions for the simultaneous delivery of these three components for bone fusion applications.^7^ To this end, several orthopedic companies have commercialized allogenic bone grafts, which contain live cells, known as cellular bone matrices (CBM).

Relative to their non‐cellular counterparts such as demineralized bone matrices (DBM), the benefit of maintaining viable bone‐forming cells in CBMs is not universally acknowledged.^8^ For example, it is not clear if the living cells can survive under low oxygen tension and nutrient deprivation conditions after implantation.^7^ On the other hand, bone allografts loaded with MSCs have been shown to produce more bone formation compared with non‐MSC controls through long‐term follow‐ups of 20 patients subjected to acetabular grafting during hip surgery.^9^ Although the superiority of viable cell incorporation into graft materials is still being debated, CBMs presently account for more than 17% of all bone grafts and substitutes used for fusion, non‐union, and fracture repair procedures.^7^, ^10^ These products vary in their method of preparation, donor status, carrier matrix, as well as cell viability, number and concentration, which may lead to variability in their clinical performance. Considering their important role in current clinical practices, it is necessary to offer spinal surgeons an evidence‐based guide to choosing the appropriate graft type for improved quality of patient care.

Various commercially‐available CBMs have been individually evaluated in many clinical studies. Osteocel Plus led to a fusion rate ranging from 87.0% to 92.3% at up to 12 months in procedures like lateral lumbar interbody fusion (XLIF), anterior lumbar interbody fusion (ALIF), and anterior cervical discectomy and fusion (ACDF).^11^, ^12^, ^13^, ^14^, ^15^ An overall fusion rate of 89.4% and 93.5% at 12 months was observed for Trinity Evolution in a prospective clinical trial of patients undergoing single‐ and two‐level ACDF, respectively.^16^, ^17^ Similarly, a retrospective clinical evaluation of 43 patients undergoing one‐ and two‐level posterolateral lumbar arthrodesis with decompressive laminectomy showed the use of Trinity Evolution led to a fusion rate of 90.7% at 12 months.^18^ In a ViviGen study, Divi et al report 100% radiographic evidence of fusion as well as improvements in visual analog scale (VAS) and neck disability index (NDI) scores in a retrospective case series of 21 patients undergoing multilevel anterior and posterior cervical fusion.^19^ However, it is impossible to compare these fusion rates across studies due to different surgical procedures used, patient characteristics and population size, and fusion criteria. In addition, most of these studies were uncontrolled and were conducted using a non‐randomized retrospective analysis, providing less convincing evidence regarding their comparative effectiveness.

As an alternative to the costly clinical approach involving human subjects, an athymic rat posterolateral spinal fusion model has been increasingly used to assess the effectiveness of a particular treatment for spine fusion due to its low cost and uncomplicated control of variables.^20^, ^21^ Other advantages of this rat model include ease of animal handling, similar spinal morphology to humans, a simple surgical technique, minimal immunologic response to human tissue and low complication rates. Using this model, the ability of three CBMs to produce a stable spine fusion at the L4‐5 level was successfully compared previously.^22^

The present study aims to compare the effectiveness of six different CBMs in an athymic rat model of posterolateral spinal fusion, due to their wide use in‐patient treatment and the fact that no information regarding their relative effectiveness is available. Although animal results cannot be directly translated to human clinical outcomes, this study aims to provide some evidence and discussion for the appropriate use of CBMs in patient care.

## 2 MATERIALS AND METHODS

### 2.1 Blinding and randomization procedures

In order to avoid any bias, the following randomization and blinding procedures were adopted throughout the study. First, animal surgeries of the seven graft types were randomly placed into the surgery schedule. In addition, two surgeons (C.L. and P.P.) were randomly assigned to the surgery and not aware of the allograft name on the day of surgery. The fact that one surgeon could be more familiar with this rat model than the other may lead to personnel bias. This was mitigated by having the surgeries for each allograft split between the two surgeons. Second, careful anonymity of implant type was kept during the data collection and analysis. Manual palpation was performed by three blinded reviewers (C.L., P.P. and J.Y.) with no information of graft type at the time of evaluation. MicroCT scans and histological sections were analyzed by two independent parties (ImageIQ, Cleveland, Ohio and Histion LLC, Everett, Washington), respectively. Both of them received only the coded samples with no allograft information. The graft information was first un‐blinded only after the analysis was completed. Thus, every effort was made to ensure a high‐quality study with the most objective scientific evidence for the relative effectiveness of the tested allograft types.

### 2.2 Cellular bone matrices

A total of six types of commercial allografts were evaluated (Table 1). The graft types included Trinity ELITE® allograft (Orthofix Medical, Lewisville TX), ViviGen® (DePuySynthes, West Chester, PA), Cellentra® (Zimmer Biomet, Warsaw, IN), Osteocel® Pro (NuVasive, San Diego CA), Bio4® (Stryker, Kalamazoo, MI) and Map3® (RTISurgical, Marquette, MI). Each cell‐based implant was stored, thawed and prepared in the manner laid out in the manufacturer's instructions for use and all implantations occurred within the manufacturers' time allowance for use after preparation. Syngeneic iliac crest bone from donor rats was used as a control group, with a separate donor animal per experimental animal in the control group. The inclusion of this control group created seven total graft types for implantation. To allow for lot‐to‐lot variability of the allografts, three different lots of each implant type were procured and each lot was used to implant five rats, for a total of 15 rats per allograft type.

**Table 1:** Cellular bone matrices evaluated in this study and their basic characteristics^a^

Graft name	Vendor	Components	Cell count	Cell viability^b^
Trinity ELITE	Orthofix Medical	Cancellous bone containing viable cells and demineralized bone	≥500 000 cells/cc, of which >100 000 cells/cc are osteogenic cells	≥70%
Vivigen	DePuy Synthes	Corticocancellous chips containing lineage committed bone cells and demineralized bone particulate	>16 000 cells/cc	96%
Cellentra	Zimmer‐Biomet	Cancellous bone containing viable cells and demineralized cortical bone	≥ 250 000 cells/cc in the cancellous tissue	≥70%
Osteocel Pro	NuVasive	Cryopreserved viable cancellous matrix and ground demineralized bone matrix	Average of 3 million cells/cc	>85% on average
Bio4	Stryker	A cryopreserved viable bone matrix product that contains native matrix, endogenous osteoblasts and MSCs, and osteoinductive and angiogenic growth factors	On average, ≥ 600 000 cells/cc	≥70%
Map3	RTI Surgical	Cortical cancellous bone chips, demineralized bone matrix and MAPC‐class cells^c^	≥50 000 viable cells/cc of implant	Not available

a All information was acquired from the manufacture website, product package insert and brochure.

b After the manufacture recommended thawing procedures.

c Multipotent Adult Progenitor (MAPC)‐class cells were obtained through the manufacture proprietary procedures.

### 2.3 Athymic rat fusion model

The animal use protocol was approved by the Institutional Animal review Board at Oregon Health and Science University, and complied with the following NIH guidelines.^23^ Male athymic rats were obtained from an institutional colony and aged until 250 to 300 g (8‐10 weeks of age). Implantation surgeries for the seven graft types were randomly assigned to two surgeons, who were completely blinded to experimental groups. To minimize bias, none of the graft types was implanted by only one surgeon. Rats were anesthetized with buprenorphine and isofluorane inhalational anesthetic. After anesthesia and skin preparation, a single posterior midline longitudinal skin and subcutaneous incision was performed. Subsequently, bilateral longitudinal paraspinal myofascial incisions were made to expose the transverse processes and intertransverse membranes at the L4 to 5 level. The processes were decorticated with periosteal elevator and 0.3 cc of bone graft was implanted bilaterally. The skin and fascial incisions were closed with 4‐0 absorbable suture. One rat from the Vivigen group died during the recovery period post‐surgery. A replacement surgery was carried out the following week. Post‐surgery, the rats were allowed free cage movement, food and water ad libitum. Rats were euthanized at 6 weeks and lumbar spines harvested for further manual palpation, X‐ray and microCT evaluation.

### 2.4 Manual palpation

Manual palpation has been validated as a sensitive and specific method of assessing fusion in this model.^20^, ^21^, ^24^ Spines were evaluated for stable fusion by manual palpation and scored as either fused or not fused. Fusion was defined as the lack of motion between L4 and L5 vertebrae. The evaluation was carried out by three independent surgeons blinded to the experimental groups. If a fused score was given by at least two out of three surgeons, that spine was considered as fused.

### 2.5 Radiographic analysis

**Figure 1:**
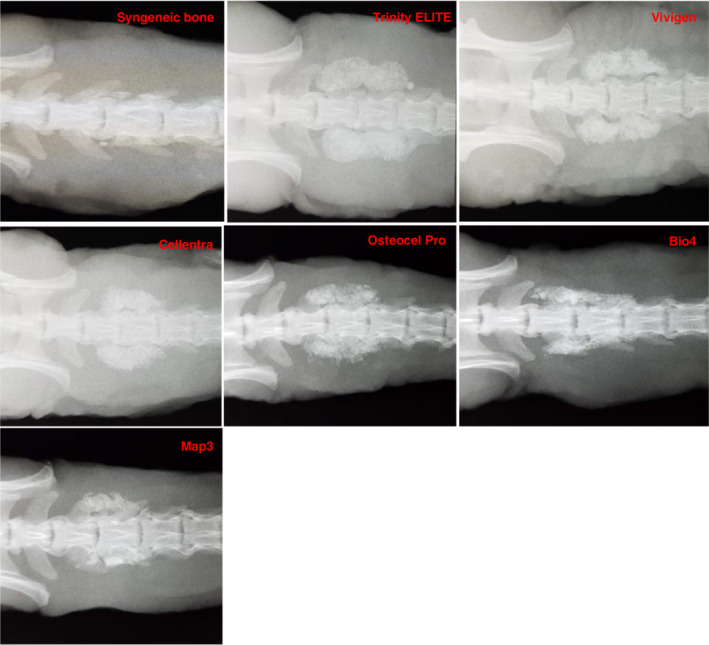
Representative X‐ray images of full fusion mass at week 6

Plain anteroposterior radiographs (Faxitron) were taken at 6 weeks (Figure 1). They were scored by a single‐blinded observer according to a 3‐point scale: 0 = absence of continuous fusion mass between transverse processes on either side, 1 = presence of continuous fusion mass between transverse processes on one side only and 2 = presence of continuous fusion mass between transverse processes on both sides.

### 2.6 MicroCT analysis

Within 48 hours of surgery, an in vivo microCT scan (59 μm, Perkin Elmer Quantum FX scanner) was performed for each animal to record the baseline/day 0 value of the total bone volume (TBV) and bone mineral density (BMD) of the region of interest (ROI). After the first microCT scan, rats were subjected to normal cage activities until sacrifice at week 6. A second microCT scan was subsequently taken to track TBV and BMD changes from the baseline. The raw microCT data were processed and analyzed by an independent contract research organization (ImageIQ, Cleveland, Ohio) blinded to the experimental conditions. Anonymity of implant type was rigorously kept to avoid any bias.

For the TBV and BMD analysis, ROI was defined as the region from the middle of L3 to the inferior endplate of L5, because bone outgrowth beyond L4 towards L3 was observed. The same ROI was used for both day 0 and week 6 analyses. Due to the difficulty in separating rat spine vertebrae from original allograft material or newly formed bone at both time points, rat vertebrae were not excluded from the TBV and BMD analysis. The percentage of the TBV increase was calculated by dividing the TBV differences between day 0 and week 6 by its baseline TBV at day 0. These percent changes were plotted in Figure 4. For the BMD analysis, two phantom rods at a density of 0.25 and 0.75 g/cm^3^ CaHA were used to calibrate the microCT scans.

### 2.7 Histology

Following explantation and assessment of fusion, one out of five rat spines per lot of each allograft type was randomly chosen and fixed in 10% formalin, relabeled and coded with a combination of letters and numbers to mask any identifying information about the graft. A total of 21 coded spine samples from the seven experimental groups (three per experimental group) were sent to an independent CRO (Histion LLC) for histological analysis. At Histion LLC, they were bisected through the midline in a sagittal plane to produce right and left fusion masses, and then embedded in paraffin. Five sections (4 μm) were taken from each fusion mass. Two of these sections were stained with hematoxylin and eosin (H&E) stain, while another two were stained with toluidine blue. Representative H&E samples were selected and evaluated for bony remodeling as well as evidence of infection, inflammation and adverse tissue reaction (Figure 7).

The two toluidine‐blue‐stained sections taken from each fusion mass were evaluated microscopically by an experienced reviewer blinded to the treatment groups. All sections were scored using a semi‐quantitative scheme (Table 2) for woven bone, lamellar bone, bone maturation and total bone & bone marrow.^25^ A preliminary evaluation of the presence of woven bone for all seven implant types indicated that for all the sections, the woven bone occupied less than 25% of the implant area. To improve the sensitivity of this semi‐quantitative scoring approach, a smaller scoring spread was used for the woven bone (Table 2). The scores from two sections of each fusion mass were averaged together and treated as one independent observation in the statistical analysis.

**Table 2:** Semi‐quantitative scoring scheme for toluidine blue stain

Woven bone	Score	Bone maturation	Score
10%‐25%	2	>75% lamellar bone	4
0%‐10%	1	50%‐75% lamellar	3
None	0	25%‐50% lamellar	2
—	—	<25% lamellar bone	1

Lamellar bone	Score	Total bone/bone marrow	Score
75%‐100%	4	75%‐100%	4
50%‐75%	3	50%‐75%	3
25%‐50%	2	25%‐50%	2
0%‐25%	1	0%‐25%	1
None	0	None	0

*Note*: Each bone parameter was evaluated based on their percentage occupancy within the implant area.

### 2.8 Statistical methods

Fisher's exact test was used for the statistical comparison of the manual palpation scores with statistical significance set at *P* < .05. For the TBV, BMD and semi‐quantitative histological analyses, data were represented as mean ± SD. Statistical comparisons were performed using the Student's *t* test with significant difference set at *P* < .05. The relationship between the new bone formation at week 6 and initial bone volume at day 0 was assessed using the linear regression model with the least squares. *F* test was used to test the overall significance of this model.

## 3 RESULTS

### 3.1 Manual palpation

Fusion was assessed via manual palpation at the L4 to 5 motion segment and scored as fused or not fused (Table 3). By this method, the syngeneic bone control group had a fusion rate of five of 15 (33%). By comparison, 11 of 15 rats in the Cellentra group, eight of 15 in the Trinity ELITE group and two of 15 in the Vivigen group had achieved fusion. None of the rats in the OsteoCel Pro, Bio4 and Map3 groups produced fusion as assessed by manual palpation. Statistical analysis showed that the Trinity ELITE and Cellentra groups had significantly higher fusion rates than all the other cell‐based allografts, but were not different from each other.

**Table 3:** Manual palpation scores

Graft type	Number fused	Percent fused (%)	Number fused by lot		
			Lot 1	Lot 2	Lot 3
Syngeneic bone	5/15	33.3	—	—	—
Trinity ELITE*	8/15	53.3	1/5	3/5	4/5
Vivigen	2/15	13.3	0/5	1/5	1/5
Cellentra	11/15	73.3	3/5	3/5	5/5
OsteoCel Pro	0/15	0	0/5	0/5	0/5
Bio4	0/15	0	0/5	0/5	0/5
Map3	0/15	0	0/5	0/5	0/5

*Statistical comparison: Trinity ELITE allograft vs Syngeneic bone or Cellentra: p > 0.05; Trinity ELITE allograft and Cellentra vs all others except syngeneic bone: p < 0.05.

For the three allograft types that yielded stable fusions, a review of the findings within each lot showed that the numbers of stable fusions varied by donor source (Table 3). For Trinity ELITE, the numbers were one of five, three of five and four of five, while those for Cellentra were three of five, three of five and five of five. Two successful fusions from Vivigen came from two different lots.

### 3.2 Radiographic analysis

**Figure 2:**
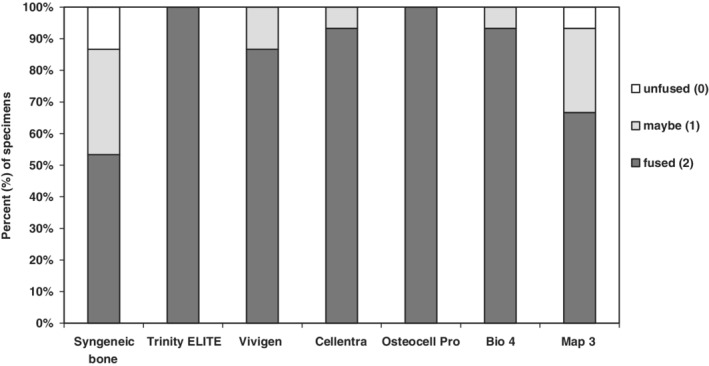
Radiographic scores of allografts 6 weeks post‐implantation in the athymic rat lumbar spine

X‐rays are taken at 6 weeks generally demonstrated that a continuous fusion mass was present on both sides for all groups (Figure 1). However, this two‐dimensional analysis often leads to overestimation of the spinal fusion rate because the bone mass at different levels along the anterior‐posterior axis is often projected on a signal plane.^26^ In addition, the mineralized bone chips in the original graft material may not be completely resorbed at week 6 through the remodeling process. Their presence may confound the radiographic fusion results as well. Trinity ELITE and OsteoCel Pro both demonstrated 100% continuous fusion mass bilaterally, while 14 of 15 (93%) of Bio4 and Cellentra samples, 13 of 15 (87%) of Vivigen samples, 10 of 15 (67%) of Map3 and 8 of 15 (53%) of samples in the control syngeneic bone group demonstrated continuous fusion mass on both sides (Figure 2).

### 3.3 MicroCT analysis

**Figure 3:**
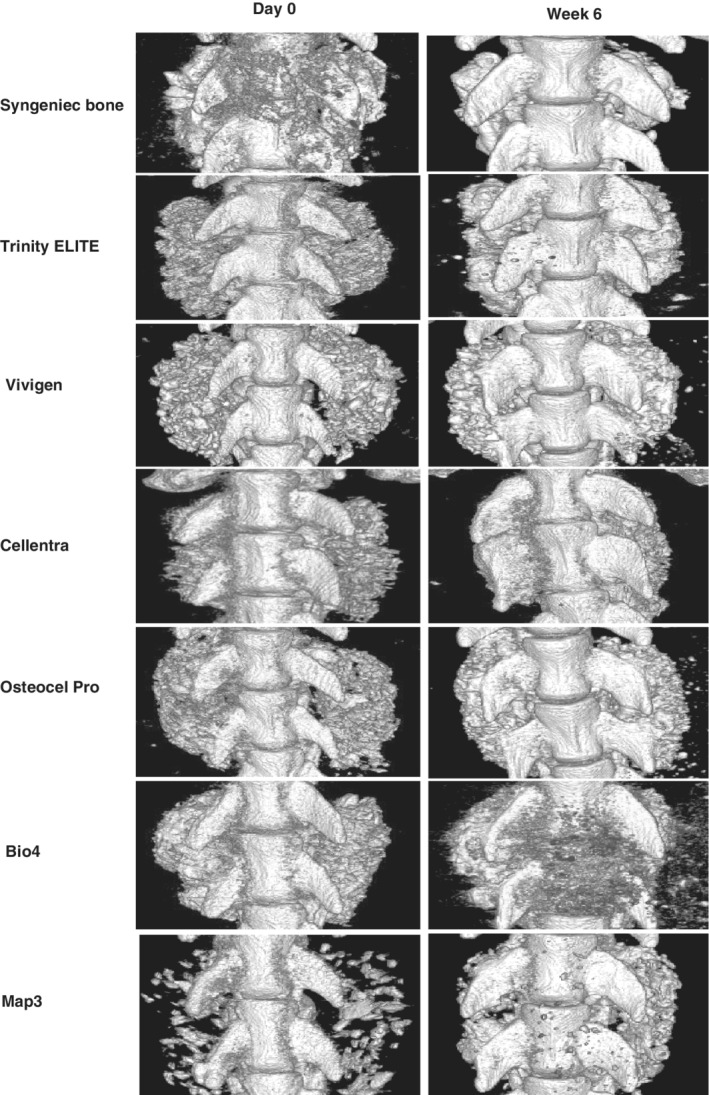
Representative three‐dimensional microCT images of full fusion mass at day 0 and week 6

Due to technical problems, the microCT scans for one Osteocel Pro sample at day 0 and one Vivigen sample at week 6 failed. These two rat spines were excluded from the analysis. The 3D microCT images showed that the implant materials that were placed bilaterally at L4 to 5 levels at day 0 had remodeled over the 6‐week period to form a fusion mass for all groups (Figure 3).

**Figure 4:**
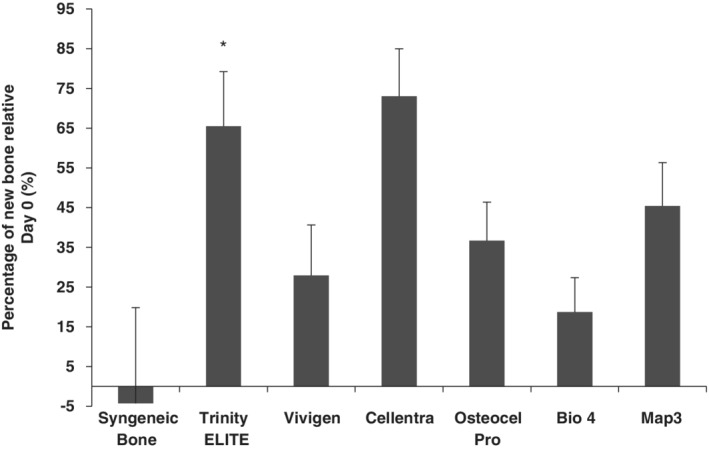
Percentage of new bone formation over a 6‐week period after implantation. *Statistical comparison: Trinity ELITE allograft vs Cellentra *P* = .12; Trinity ELITE or Cellentra allograft vs all others *P* < .05; syngeneic bone vs all other CBMs *P* < .05

All of the allograft groups showed an increase in bone volume over the 6‐week period (Figure 4). The Cellentra group had a 73% increase compared with baseline and the Trinity ELITE group had a 65% increase relative to baseline. While there was not a statistically significant difference between these two groups (*P* = .12), the percentage increase in both groups was significantly greater compared with other groups (*P* < .05) (Figure 4). The percentage increases for Vivigen, Osteocel Pro, Bio4 and Maps are 29%, 37%, 19% and 45%, respectively. In the syngeneic bone group, the changes in bone volume varied from −35% to +48%, leading to a large SD for this group. On average, the whole group had slight bone loss over the 6‐week period.

**Figure 5:**
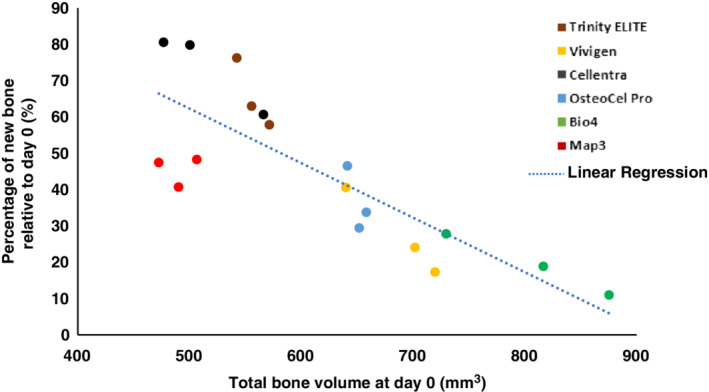
Percentage of new bone formation from each allograft lot is inversely related to its total bone volume at day 0. Each dot represents one lot. Linear Regression, *R* ^2^ = 0.67; *P* value of *F* test <.0001

Percentages of new bone formation were calculated on a lot basis, and plotted against the total bone volume at day 0 of the same lot in Figure 5. Both the new bone formation (y‐axis) and baseline bone volume (X‐axis) varied by lot for all allografts. Since the same volume (0.3 cc) of allografts was implanted at each side of the spine at day 0, the baseline bone volume variations between graft types were likely due to differences in composition, microstructure, biochemical nature and spatial arrangement of the components. Figure 5 also showed a trend that amount of new bone formation is inversely related to the total bone volume at day 0 on a lot basis across graft types except Map3.

**Figure 6:**
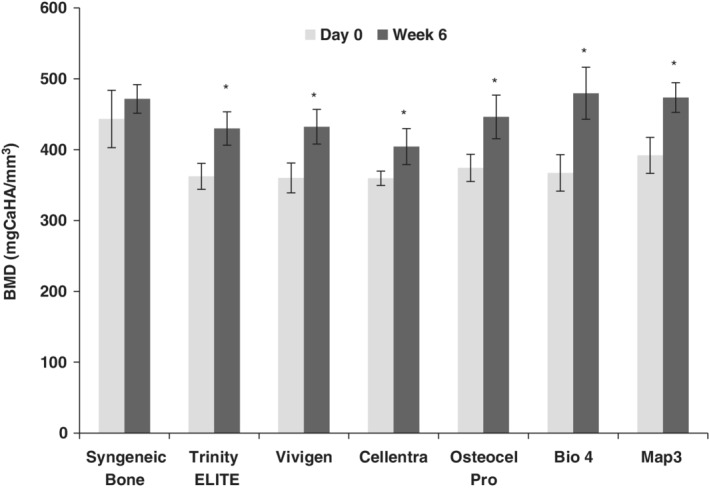
Bone mineral density (BMD) at day 0 and week 6. A significant BMD increase relative to day 0 was indicated by asterisk *P < .05

At day 0, all commercial allografts (except Map3) had a statistically similar but significantly lower bone mineral density (BMD) than syngeneic bone (Figure 6). This was expected, as all of the allografts contained a demineralized component thus resulting in a lower BMD for the region of interest relative to syngeneic bone.

Six weeks after the implantation, significant BMD increases were seen across the CBM groups with Bio4 having the greatest BMD increase of 112 mgCaHA/mm^3^, although BMD for Cellentra was statistically lower compared to all other implants. The increases for Trinity ELITE (68 mgCaHA/mm^3^), Vivigen (71 mgCaHA/mm^3^), OsteoCel Pro (75 mgCaHA/mm^3^) and Map3 (82 mgCaHA/mm^3^) were statistically similar, but statistically higher than those of Cellentra (45 mgCaHA/mm^3^) and syngeneic bone (28 mgCaHA/mm^3^).

### 3.4 Histologic analysis

**Figure 7:**
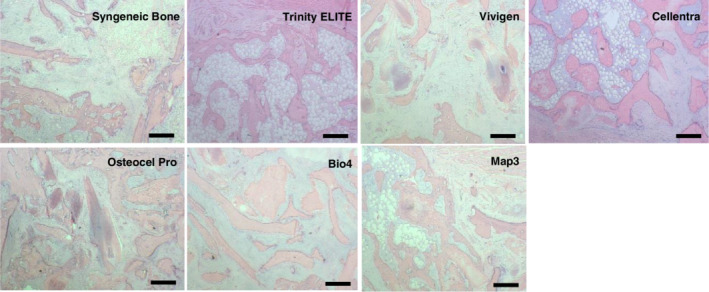
Representative hematoxylin and eosin stained histological sections of implants 6 weeks post‐implantation. Scale bar 500 μm

Six weeks after the implantation, H&E staining did not reveal any signs of infection for all of the seven implant groups (Figure 7). With respect to inflammation, six allografts showed similar but minimal cellular infiltration, which was composed of macrophages and giant cells associated with residual implant allograft particles. Fibrous tissue was also evident in all groups, located between pieces of residual implant material.

**Figure 8:**
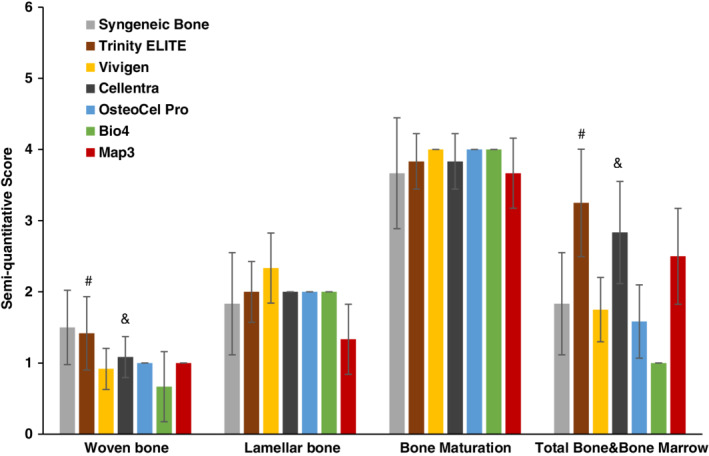
Semi‐quantitative histopathological scores 6 weeks post‐implantation in the athymic rat lumbar spine. ^#^Statistical comparison: Trinity ELITE allograft vs Cellentra *P* = .06 (woven bone) and 0.18 (total bone and bone marrow); Trinity ELITE allograft vs all others in woven bone or total bone and bone marrow analyses: *P* < .05. ^&^Statistical comparison: Cellentra vs Vivigen, or OsteoCel Pro, or Map3 *P* > .05 but Bio4 *P* = .02 in woven bone; for total bone and bone marrow, Cellentra vs all others except Trinity ELITE and Map3 *P* < .05

Both H&E and toluidine blue‐stained sections demonstrated bone formation and reconstitution of marrow elements for all graft groups, but to a significantly different degree. To quantitatively capture these differences, the toluidine blue staining sections were scored in terms of their woven bone, lamellar bone, bone maturation and total bone and bone marrow according to the criteria in Table 2. These scores were analyzed and shown in Figure 8. For the woven bone scores, syngeneic bone was statistically higher than all other groups (*P* < .05) except Trinity ELITE (*P* = .7). This score for Trinity ELITE was statistically similar to that for Cellentra (*P* = .06), but higher than the rest of CBM groups (*P* < .05). For the lamellar bone scores, Vivigen was not significantly different from Trinity ELITE (*P* = .09) or syngeneic bone (*P* = .06), but greater than Cellentra, OsteoCel Pro, Bio4 and Map3 (*P* < .05). For bone maturation, Map3 had a significant lower score than Vivigen, OsteoCel Pro and Bio4. No other significant differences were seen between other pair comparisons. There was no statistical difference between total bone and bone marrow scores of Cellentra and Trinity ELITE (*P* = .18). However, Trinity ELITE was significantly higher than the rest of the groups (*P* < .05). Cellentra was significantly higher than Vivigen, OsteoCel Pro and Bio4 (*P* < .001), but not Map3 (*P* = .25).

## 4 DISCUSSION

This is the first study to broadly evaluate the fusion potential of commercially available CBMs in an athymic rat model. The fusion rate in this rat posterolateral spinal fusion model is highly dependent on the timing of assessment.^20^ A meta‐analysis summarizing 26 studies using this rat model found a pooled fusion rate of 38.1% at 6 weeks regardless of graft types.^20^ The authors further suggested that 6 weeks could be a good time point for the negative control. In another rat posterolateral study, autograft implants alone led to a 30% fusion rate at 6 weeks.^21^ In the present study, the syngeneic bone group at week 6 yielded a fusion rate of 33.3% in the manual palpation assessment, which is well within the expected range based on the historical results from this animal model. The syngeneic bone group was included in this study as a reference to detect any improved and/or earlier fusion with use of the CBMs.

Both X‐ray and manual palpation were used to evaluate the fusions in this study. The advantages of using plain X‐ray for spinal fusion assessment include easy accessibility, low cost and low radiation dose compared to computed tomography.^27^ Moreover, plain X‐ray is a standard technique in the current clinical practices to check fusion, factures, etc. In our study, this technique was included to mimic the clinical scenario for fusion assessment. However, this modality has limited ability to quantify bone formation and to determine presence or absence of fusion.^26^ A majority of the rats in the present study established bone bridging on at least one side for all the implant groups, which is not well aligned with the manual palpation scores. Manual palpation is a more accurate method of fusion evaluation in this rat model. It has a better accuracy in assessing actual lack of motion between vertebras^26^ and is considered as the gold standard of fusion assessment.^20^ Based on manual palpation, Trinity ELITE and Cellentra implants resulted in significantly greater fusion rate compared to all other allograft types.

To better understand the nature of fusion, the TBV and BMD of the ROIs were measured. One limitation for these microCT results is that rat vertebrae were not excluded from the analysis. Thus, the reported TBV is a sum of vertebrae, residual implant pieces and newly formed bone at 6 weeks. In order to compare the TBV increase solely attributed to implanted materials across the groups, it has to be assumed that either the vertebrae do not significantly grow/remodel or the amounts of their growth across the experimental groups are equal. Rats of 8 to 10 weeks of age used in this study were considered to be at their skeletal maturity^28^ and did not experience significant spinal growth over a 6‐week period, making the assumption reasonable. Another limitation of the microCT is that this technique is not able to differentiate the amount of newly formed bone from the residual graft pieces. However, it is important to distinguish the new bone from the original bone since this metric provides direct evidence of the ability of the implant to promote fusion. Histologically, it is possible to obtain such differentiation since the color of the staining and the morphology of the tissue are different. Therefore, a semi‐quantitative histological evaluation was included in this study as a complementary assessment of fusions to quantify the amount of new bone and bone marrow within the ROI.

A larger TBV increase over a 6‐week period from both Trinity ELITE and Cellentra relative to other implants suggested that more bone mass was added between vertebrae to prevent the motion, which is consistent with the overall manual palpation scores (Table 3 and Figure 4). However, this is not the case for the syngeneic bone, where a fusion rate of 33.3% was coupled with slight bone loss 6 weeks post‐surgery. It should be noted that upon implantation of the graft materials, bone remodeling occurred thereafter. This remodeling process is critical for incorporation of the bone allograft to gain a bony fusion.^29^, ^30^ Under certain circumstances, bone resorption during this process becomes significant.^31^ In the case of syngeneic bone, it is likely that the originally implanted bone volume from iliac crest bone was resorbed through osteoclastic activities during the remodeling, while a lesser amount of new bone was actively deposited around the L3 to 4 and L4 to 5 processes to immobilize the spine. In fact, histological analysis showed that Trinity ELITE, Cellentra, and syngeneic bone had a greater amount of woven bone within the implant area at week 6 (Figure 8), indicating relatively more active bone remodeling. Consistent with a greater presence of the woven bone, Trinity ELITE and Cellentra groups had a significantly higher total bone and bone marrow score. In contrast, OsteoCel Pro, Map3 and Bio4 produced no manual palpation fusions and had lower woven bone scores, although their TBV increases over the 6‐week period (Figure 4). A lower woven bone score for these three allografts implies that their post‐surgery remodeling process and integration with the host tissue were not as strong as that for Trinity ELITE, Cellentra and syngeneic bone. Overall, evidence from multiple analyses suggested that ability of these CBMs to produce a stable fusion in this rat model differed significantly.

It is difficult to truly determine why these commercial CBMs performed differently in this animal model without knowing their preparation methods, exact composition or their physical, chemical and biological properties. In particular, there are two characteristics of CBMs, which likely vary between these commercially‐available products that are likely strongly related to successful fusions. The first characteristic is the level of osteoinductive potential, which is associated with the concentration of endogenous growth factors such as BMP‐2 in the demineralized bone component of the CBMs. Previous studies have demonstrated that demineralized bone allografts generated by different tissue processors may have considerably different BMP‐2 content^32^, ^33^ and that higher BMP‐2 levels are correlated with increasing osteoinductivity and fusion rates.^34^, ^35^, ^36^ The second characteristic is the number of viable osteogenic cells that are contained in the CBMs. While this information is not specified by all of the different tissue processors, the information that was available indicated that osteogenic cell counts can vary considerably.^7^ In addition to the number of viable cells in each graft type, it is possible that varying processing and storage conditions as well as thawing procedures could influence subsequent cell functionality and the ability of the cells to contribute to the bone healing process. Furthermore, upon implantation, cells are subjected to immune responses from the host body and microischemia conditions with low oxygen tension and nutrient deprivation.^37^ Toma et al found that only 14% of MSCs delivered into rat cremaster muscle by microcirculation were alive at 24 hours.^38^ Similarly, Degano et al found that only 37% of bone marrow‐derived MSCs implanted into a calvarial bone defect were viable after 90 days.^39^ Thus, the number of cells surviving in vivo may be very different from what was originally present before implantation.

One interesting phenomenon observed, for all allografts tested except Map3, was that TBV at day 0 is inversely related to the in vivo performance for promoting bone formation and creating stable fusion at week 6 (Table 3, Figures 4 and 5). This is true not only across the graft types but also on a lot basis within each graft type. Although a volume of 0.3 cc was implanted on each side for all grafts at day 0, their TBV as measured by microCT varies significantly between graft types. Considering that the BMD is statistically similar for all CBMs except Map3, their day 0 TBV variations were likely due to the different particle sizes of the bone chips, degree of mineralization due to processing, spatial arrangement and ratio of subcomponents, porosity and amount of soft tissues containing growth factors. These differences can affect not only the initial nutrient and oxygen supply to the implanted cells upon implantation, but also the subsequent vascular ingrowth and bone remodeling rates.^40^ Implants with appropriate amounts of void space between bone chips may have more efficient interactions with host cells and tissues, which may be the reason why Trinity ELITE and Cellentra, with the least day 0 TBV, generated the most amount of new bone after 6 weeks, while Bio4 with the most day 0 TBV exhibited the opposite effect. This bone volume relationship seems to also hold true on a lot‐by‐lot basis within each type of allograft (Figure 5). As an example, the three lots of Bio4 with a day 0 TBV of 730, 817 and 875 mm^3^, respectively, produced an inversely corresponding amount of new bone: 203, 154 and 96 mm^3^ at week 6, respectively.

A similar trend exists for the relationship between the day 0 TBV and manual palpation scores at week 6. For example, the three lots of Trinity ELITE had a bone volume of 571, 556, 542 mm^3^ at day 0, respectively. Their corresponding manual palpation scores were one of five, three of five and four of five. Pooling these results from all CBMs, it appears a graft lot with a day 0 bone volume less than 640 mm^3^ tends to have a better chance of creating a stable bony fusion in this study. However, Map3 is the only allograft type that falls out of this trend, possibly due to potentially different manufacturing procedures or graft composition. Instead of preserving viable MSCs that are endogenous to the donor bone during the manufacturing process, Map3 utilizes Multipotent Adult Progenitor‐Class Cells (MAPC) as the osteogenic viable cell source. These cells are isolated from bone marrow through proprietary procedures and combined with non‐viable donor bone at the time of surgery.

The primary goal of a spinal fusion procedure is to achieve a solid fusion.^41^ A mechanically weak fusion leads to uncontrolled motion between vertebrae, and patients may be subjected to pain, secondary surgery and decreased quality of life. One of the limitations of this study is that no biomechanical testing was conducted to evaluate the quality of the fusions. However, the ultimate force required to cause fusion fracture, stiffness and mechanical strength of fusion masses have previously been quantified to offer a comprehensive view of the fusion quality in different animal models.^42^, ^43^ In these studies, fusions with lower BMD were found to be closely related to decreased bone strength and local adverse structural alterations of the fusion mass. On the other hand, bone samples with a higher BMD have shown superior mechanical properties, such as Young's/structural modulus and compression strength.^44^ However, in the present study, there did not appear to be a positive correlation between BMD and fusion rates. Therefore, a direct biomechanical test of the fusion masses by these allografts may be beneficial in future studies.

A second limitation of this study is that only one‐time point (week 6) was used to assess the fusions. At this time point, syngeneic bone grafts led to a fusion rate around 33.3%. Historically, for syngeneic bone when utilized in this type of rat model, an assessment time‐point of 8 weeks is the threshold for a higher fusion rate.^20^ In particular, at 8 weeks, fusion rates by the syngeneic bone may be as high as 70%.^43^ Similarly, fusions by the allograft implants examined in this study may increase significantly beyond week 6 as well, although not necessarily at the same rate. The results from this study only demonstrated the superior performance by Trinity ELITE and Cellentra in establishing early fusion up to week 6 relative to other implants. It is not clear if the superiority will last further beyond the 6 week time point.

Caution should be taken while translating the current results into clinical results of human subjects. As with other animal models, the results seen in animals are not always reproducible in humans, and conclusions drawn from rodent studies may not be directly translated to human clinical use.

## 5 CONCLUSION

In summary, this study demonstrated that there is significant variation in the ability of different commercially available CBMs to produce posterolateral spinal fusion in a rat model. Trinity ELITE and Cellentra were found to have the greatest potential for successful early fusion at week 6 as measured by manual palpation and the highest percentage increase in bone volume as measured by microCT. Since the specific composition and preparation methods of each product are proprietary and undisclosed, it is not possible to explicitly determine the underlying factors that led to the differences in fusion results.

## CONFLICT OF INTEREST

This study was supported by a research grant received by B.J. and J.Y. from Orthofix Medical Inc. (Lewisville Texas) and MTF Biologics (Edison, New Jersey). J.T.R., E.I.W. and N.Z. are employees of and own stocks in Orthofix Medical Inc. E.S. and D.W. are employees of MTF Biologics. Since MTF Biologics and Orthofix Medical Inc. are the manufacturer and distributor of the Trinity ELITE allografts, the following procedures were taken to mitigate the potential conflict of interest: (a) animal surgeries were performed with rigorous randomization and blindness, including surgeries of the seven graft types being randomly placed into the surgery schedule; (b) two surgeons were randomly assigned to the surgery and not aware of the allograft name on the day of surgery; the surgeries for each allograft were split between the two surgeons; (c) careful anonymity of implant type was kept during the data collection and analysis. Manual palpation was performed by three independent reviewers with no information of graft type at the time of evaluation. MicroCT scans and histological sections were analyzed by two independent parties, who received only the coded samples with no allograft information; (d) per the research agreement between the sponsor (Orthofix) and Principal Investigator (OHSU), the Principal Investigator has the right to independently publish any research findings from this study based on their academic judgment and discretion.

## AUTHOR CONTRIBUTIONS

The research was funded by a research grant to B.J. from Orthofix. J.T.R., E.I.W., N.Z., D.W., E.S., J.Y. and B.J. developed the experimental protocols and procedures. C.L. and P.P. did the animal surgeries, manual palpation, X‐ray analysis, and microCT scans. E.S., D.W. and N.Z. prepared the allograft samples for the surgeries. N.Z. and E.I.W. worked with the ImageIQ LLC and Histion LLC, analyzed the microCT images and histological results. C.L. and N.Z. drafted the manuscript. All authors reviewed the manuscript.

## Data Availability

All data and materials in this study will be available upon request by contacting the corresponding authors.
